# HMEC: A Heuristic Algorithm for Individual Haplotyping with Minimum Error Correction

**DOI:** 10.1155/2013/291741

**Published:** 2013-01-28

**Authors:** Md. Shamsuzzoha Bayzid, Md. Maksudul Alam, Abdullah Mueen, Md. Saidur Rahman

**Affiliations:** ^1^Department of Computer Science, University of Texas at Austin, Austin, TX 78712, USA; ^2^Department of Computer Science, Virginia Tech, Blacksburg, VA 24060, USA; ^3^Department of Computer Science and Engineering, Univerity of California, Riverside, CA 92521, USA; ^4^Department of Computer Science and Engineering, Bangladesh University of Engineering and Technology, Dhaka 1000, Bangladesh

## Abstract

Haplotype is a pattern of single nucleotide polymorphisms (SNPs) on a single chromosome. Constructing a pair of haplotypes from aligned and overlapping but intermixed and erroneous fragments of the chromosomal sequences is a nontrivial problem. Minimum error correction approach aims to minimize the number of errors to be corrected so that the pair of haplotypes can be constructed through consensus of the fragments. We give a heuristic algorithm (HMEC) that searches through alternative solutions using a gain measure and stops whenever no better solution can be achieved. Time complexity of each iteration is *O*(*m* ^3^ *k*) for an *m* × *k* SNP matrix where *m* and *k* are the number of fragments (number of rows) and number of SNP sites (number of columns), respectively, in an SNP matrix. Alternative gain measure is also given to reduce running time. We have compared our algorithm with other methods in terms of accuracy and running time on both simulated and real data, and our extensive experimental results indicate the superiority of our algorithm over others.

## 1. Introduction

 A single DNA molecule is a long chain of nucleotides (base pairs). There are four such nucleotides which are represented by the set of symbols {*A*, *T*, *G*, *C*}. It is generally accepted that genomes of two humans are almost 99% identical at DNA level. However, at certain specific sites, variation is observed across the human population which is commonly known as *“single nucleotide polymorphism”* and abbreviated as “SNP” [1]. The nucleotide involved in a SNP site is called *allele*. If a SNP site can have only two nucleotides, it is called *biallelic*. If it can have more than two alleles it is called a *multiallelic* SNP [2]. From now on, we will consider the simplest case where only bi-allelic SNPs occur in a specific pair of DNA.

The single nucleotide polymorphism (SNP) is believed to be the most widespread form of genetic variation [3]. The sequence of all SNPs in a given chromosome is called haplotype. Haplotyping an individual deals with determining a pair of haplotypes, one for each copy of a given chromosome. A chromosome is a complicated structure of a DNA molecule bound by proteins. This pair of haplotypes completely define the SNP fingerprints of an individual for a specific pair of chromosomes. Given the two sequences of bases, haplotyping is straight forward and just needs to iterate through both the sequences and remove all the common alleles from them. But haplotyping becomes difficult when we want to construct haplotypes from sequencing data for higher reliability. Sequencing data for a genome does not contain the complete sequences of bases for a specific chromosome, rather it provides a set of fragments of arbitrary length for the whole genome. Therefore, the actual problem of haplotyping is to find two haplotypes from the set of overlapping fragments of both the chromosomes, where fragments might contain errors and it is not known which copy of the chromosome a particular fragment belongs to.

The problem of haplotyping has been studied extensively. The general minimum error correction (MEC) problem was proved to be NP-hard [4]. It was also proved to be NP-hard even if the SNP matrix is gapless using a reduction from the MAX-CUT problem [1]. A method based on genetic algorithm has been proposed to solve this problem [5]. Several heuristic methods have also been proposed to find haplotypes efficiently. HapCUT [6] and ReFHap [7] are two of the most accurate algorithms in this regard.

In this paper, we give a heuristic algorithm for individual haplotyping based on minimum error correction. The complexity of each iteration is *O*(*m* ^3^ *k*) for an SNP matrix of dimension (*m*, *k*). The algorithm is inspired from the famous Fiduccia and Mattheyses (FM) algorithm for bipartitioning a hypergraph minimizing the cut size [8]. Extensive simulations indicate that HMEC outperforms the genetic algorithms of Wang et al. [5] in terms of both reconstruction rate and running time, and it has better (in most cases) or comparable accuracy and significantly smaller running time than that of HapCUT [6], which is the most accurate heuristic algorithm available. We also compared HMEC with some other algorithms such as SpeedHap [9], FastHare [10], MLF [11], 2 distance MEC [12], and SHR-3 [13] using the HapMap-based instance generator and comparison framework [14, 15].

The rest of the paper is organized as follows. In Section 2, we present some definitions and preliminary ideas. In Section 3, we present our algorithm for individual haplotyping. We describe the data structure and complexity of our algorithm in Section 4. We report on an extensive performance study evaluating HMEC with other available techniques in Section 5. Finally, we conclude in Section 6 by suggesting some future research directions. An earlier version of this paper was accepted for presentation at BMEI 2008 [16].

## 2. Preliminaries

 In this section, we give some definitions and preliminary ideas.

Let *S* be the set of *k* bi-allelic SNP sites. Let *F* be the set of *m* fragments produced from two copies of the chromosome. Each fragment contains information of nonzero number of SNPs in *S*. Because the SNPs are bi-allelic, let the two possible alleles for each SNP site be 0 and 1, where they can be any two elements of the set {*A*, *T*, *G*, *C*}. Since all the nucleotides are the same at the sites other than SNP sites, we can remove these extraneous sites from all the fragments and consider the fragments as the sequences of the SNP sites only. Thus each fragment *f* ∈ *F* is a string of symbols {0,1, −} of length *k* where “−” denotes an undetermined SNP which we call a *hole*. All the fragments can be arranged in an *m* × *k* matrix *M* = {*M*_*ij*_}, *i* = 1,…, *m*, *j* = 1,…*k*, where row *i* is a fragment from *F* and column *j* is a SNP from *S*. This matrix is called the SNP matrix as follows

(1) |−−−−1101−−−−−−−−−−−−−−−−−0001110101−−−−−11010010011−−−−−−−−−−−−10100−−−010−−−−−−−−−−−−−−−1011010101101011−−−−−−−−−−01011|

The consecutive sequence of “−”s that lies between two nonhole symbols is called a *gap*. A *gapless* SNP matrix is the one that has no gap in any of the fragments. In (1), the first, second, and third rows have no gaps while each of the fourth and sixth rows has one gap.

A SNP matrix *M* = 〈*M*_1_, *M*_2_,…, *M*_*m*_〉 can be viewed as an ordered set of *m* fragments where a fragment *M*_*i*_ = 〈*M*_*i*1_, *M*_*i*2_,…, *M*_*ik*_〉 is an ordered set of *k* alleles. A fragment *M*_*i*_ is called to *cover* the *j*th SNP if *M*_*ij*_ ∈ {0,1} and called to *skip* the *j*th SNP if *M*_*ij*_ = −. Let *M*_*s*_ and *M*_*t*_ be two fragments. The distance between two fragments, *D*(*M*_*s*_, *M*_*t*_), is defined as the number of SNPs that are covered by both of the fragments and have different alleles. Hence,

(2) $$\begin{array}{c} D\left(M_{s},M_{t}\right)=\sum_{j=1}^{k}d\left(M_{sj},M_{tj}\right), \end{array}$$

where *d*(*x*, *y*) is defined as

(3) $$\begin{array}{c} d\left(x,y\right)=\{\begin{array}{cc} 1, & \text{if }x\neq -\;\text{and}\;y\neq -\;\text{and}\;x\neq y;\\ 0, & \text{otherwise}. \end{array} \end{array}$$

In (1), the distance between the second and the third fragment is two, as they differ in the seventh and ninth SNP sites (columns).

**Figure 1:**
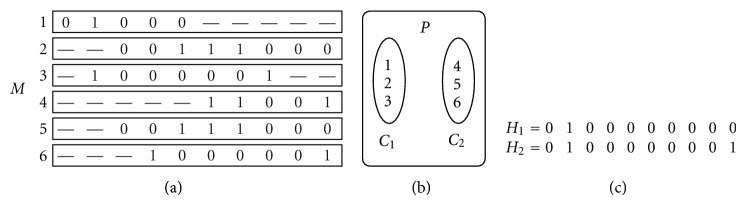
SNP matrix and its partition.

Two fragments *M*_*s*_ and *M*_*t*_ are said to be *conflicting* if *D*(*M*_*s*_, *M*_*t*_) > 0. Let *P*(*C*_1_, *C*_2_) be a *partition* of *M*, where *C*_1_ and *C*_2_ are two sets of fragments taken from *M* so that *C*_1_⋃*C*_2_ = *M* and *C*_1_⋂*C*_2_ = *ϕ* [5]. In Figure 1(b), an arbitrary partition corresponding to the SNP matrix of Figure 1(a) is shown. A SNP matrix *M* is an *error-free* matrix if and only if there exists a partition *P*(*C*_1_, *C*_2_) of *M* such that for any two fragments *x*, *y* ∈ *C*_*i*_, *i* ∈ {1,2}, *x* and *y* are non-conflicting, that is, *D*(*x*, *y*) = 0. Such a partition is called an error-free partition. The partition in the Figure 1(b) is not error free since *D*(*M*_1_, *M*_2_) > 0 in *C*_1_ and *D*(*M*_5_, *M*_6_) > 0 in *C*_2_. For an error-free SNP matrix, a haplotype *H*_*i*_, *i* ∈ {1,2} is constructed from its corresponding fragment class *C*_*i*_ using the following formula:

(4) $$\begin{array}{c} H_{ij}=\{\begin{array}{cc} 1, & \text{if}\;\text{at}\;\text{least}\;\text{one}\;\text{fragment}\;\text{in}\;C_{i}\;\text{has}\;\text{a}\;1\;\text{in}\;j\text{th}\;\text{SNP};\\ 0, & \text{if}\;\text{at}\;\text{least}\;\text{one}\;\text{fragment}\;\text{in}\;C_{i}\;\text{has}\;\text{a}\;0\;\text{in}\;j\text{th}\;\text{SNP};\\ -, & \text{if}\;\text{all}\;\text{the}\;\text{fragments}\;\text{in}\;C_{i}\;\text{skips}\;j\text{th}\;\text{SNP}; \end{array} \end{array}$$

where *C*_*i*_ is called the defining class of haplotype *H*_*i*_, and *H*_*ij*_, where  *i* ∈ {1,2} and *j* = 1,…*k*, denotes the *j*th element of the haplotype *H*_*i*_.

We now describe the general minimum error correction problem. If a matrix *M* is *not* error-free, there will be no error-free partition *P*. For such a matrix *M*, there will be at least one conflicting pair of fragments in each of the classes for all possible partitions. Therefore it is impossible to construct a haplotype that is non-conflicting with all the fragments in its defining class of fragments. If we are given a partition *P*(*C*_1_, *C*_2_) and two haplotypes *H*_1_ and *H*_2_ constructed from *P* then the number of errors *E*(*P*) that needs to be corrected can be calculated by the following formula:

(5) $$\begin{array}{c} E\left(P\right)=\sum_{i=1}^{2}\sum_{f\in C_{i}}D\left(f,H_{i}\right). \end{array}$$

The MEC problem asks to find a partition *P* that minimizes the error function *E*(*P*) over all such partitions of an SNP matrix *M*.

## 3. A Heuristic Algorithm

 In this section, we give our heuristic algorithm based on minimum error correction which we call HMEC.

Construction of a haplotype from an erroneous class *C* requires correction of SNP values, that is, alleles, in the fragments. We want to minimize the number of error corrections. Therefore, for each SNP site, the haplotype should take the allele that is present in the majority of the fragments. Let *N*_*j*_^0^(*C*) be the number of fragments of a collection *C* that have 0 in the *j*th SNP. Similarly, *N*_*j*_^1^(*C*) defines the number of 1s [5]. Therefore, to minimize the number of errors *E*(*P*) for a specific partition *P*, the haplotype should be constructed according to the following methodology:

(6) $$\begin{array}{c} H_{ij}=\{\begin{array}{cc} 1, & \text{if}\;N_{j}^{1}\left(C_{i}\right)>N_{j}^{0}\left(C_{i}\right);\\ 0, & \text{if}\;N_{j}^{0}\left(C_{i}\right)\geq N_{j}^{1}\left(C_{i}\right)\;\text{and}\\ & \;N_{j}^{0}\left(C_{i}\right)\neq 0;\\ -, & \text{if}\;N_{j}^{1}\left(C_{i}\right)=N_{j}^{0}\left(C_{i}\right)=0; \end{array} \end{array}$$

where *i* ∈ {1,2} and *j* = 1,2,…, *k*. In Figure 1(c), two haplotypes *H*_1_ and *H*_2_, associated with the partition *P* in Figure 1(b), are constructed in this way.

**Figure 2:**
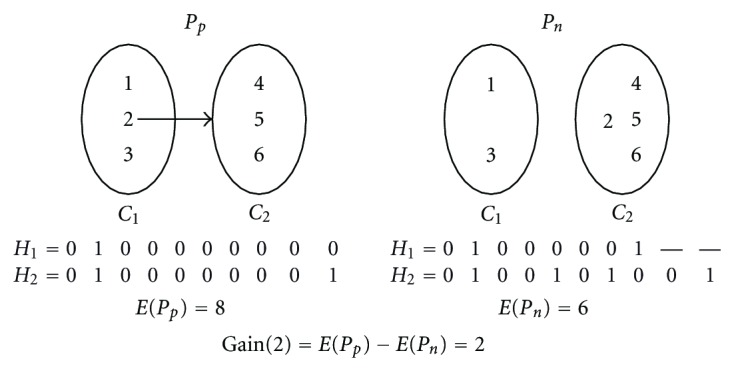
An example of Gain calculation.

We will use a heuristic search to find the best partition. This algorithm starts with a current partition *P*_*c*_ = *P*(*M*, *ϕ*) and iteratively searches a better partition. In each iteration, the algorithm performs a sequence of transfer of fragments from their present collection to the other one so that the partition becomes less erroneous. The transfer of a fragment from one collection to the other can increase or decrease the error function *E*(*P*). Let the partition before transferring a fragment *f* be *P*_*p*_ and the partition resulted is *P*_*n*_. We define the gain of the transfer as Gain(*f*) = *E*(*P*_*p*_) − *E*(*P*_*n*_).  Figure 2 demonstrates an example calculation of the gain measure. Let *F* = 〈*f*_*i*_〉, *i* = {1,2,…, *m*} be an ordering of all the fragments in a partition *P* in such a way that fragment *f*_*i*_ will precede fragment *f*_*j*_ if all the fragments before *f*_*i*_ in *F* have already been transferred to form an intermediate partition *P*_*i*_ and Gain(*f*_*i*_) ≥ Gain(*f*_*j*_) over *P*_*i*_. Hence, *P*_1_ = *P*_*c*_ at the start of each iteration. We also define the cumulative gain of a fragment ordering *F* up to the *n*th fragment as *C*Gain(*F*, *n*) = ∑_*i*=1_^*n*^Gain(*f*_*i*_). Here Gain(*f*_*i*_) = *E*(*P*_*i*_) − *E*(*P*_*i*+1_). The maximum cumulative gain, *MC*Gain(*F*) is defined as

(7) $$\begin{array}{c} MC\text{Gain}\left(F\right)=\underset{1\leq i\leq m}{\max}C\text{Gain}\left(F,i\right). \end{array}$$

In Section 4, we shall describe these terms with an example.

In each iteration, the algorithm finds the current ordering *F*_*c*_ of *P*_*c*_ and transfers only those fragments of *F*_*c*_ that can achieve the *MC*Gain(*F*_*c*_) and the fragment that is the last to be transferred is referred as *f*_max_. Thus the algorithm moves from one partition to another reducing the error function by an amount of *MC*Gain(*F*_*c*_). Please see Algorithm 1 for a basic description of HMEC. The algorithm continues as long as *MC*Gain(*F*_*c*_) > 0 and stops whenever *MC*Gain(*F*_*c*_) ≤ 0.

## 4. Data Structures and Complexity

This section deals with the data structures and the complexity of our algorithm. Here we also suggest some approximation to improve the performance of our algorithm.

First, to find *F*_*c*_ in each iteration, the algorithm repeatedly transfers the fragment that is not transferred previously in this iteration and has maximum gain over all such fragments. To accomplish this, we use a locking mechanism. At the beginning of each iteration, all the fragments are set free. The free fragment with maximum gain is found out and tentatively transferred to the other collection. After the transfer, the fragment is locked at the new collection. This tentative transfer creates the first intermediate partition *P*_1_. The algorithm then finds the next free fragment with maximum gain in *P*_1_ and transfer and lock that fragment to create the *P*_2_. Thus, free fragments are transferred until all the fragments are locked and the order of the transfer (*F*_*c*_) is stored in the log table along with the cumulative gains (*C*Gain). *MC*Gain is the maximum *C*Gain and *f*_max_ is the fragment corresponding to *MC*Gain in the log table.

After finishing all such tentative transfers, *P*_*c*_ becomes an undefined partition. To change *P*_*c*_ to the desired “current” partition of the next iteration, the algorithm checks the log to find the *MC*Gain(*F*_*c*_) and *f*_max_, and rollback the transfer of all the fragments that were transferred after *f*_max_. When the rollback completes, *P*_*c*_ becomes ready for the next iteration.

While tentatively transferring a free fragment, the algorithm needs to find the fragment with maximum gain among the free fragments (which are not yet transferred). This requires calculating gains for each of them. To calculate the Gain(*f*) = *E*(*P*_*p*_) − *E*(*P*_*n*_) for a fragment, we need to calculate two error values of two different partitions: the present intermediate partition and the next partition which will be resulted if *f* is transferred. Each of these error function requires calculation of two new haplotypes from their corresponding collections (see Figure 2). Although *E*(*P*_*p*_) and the haplotypes of *P*_*p*_ can be found from the previous transfer, calculation of *E*(*P*_*n*_) requires construction of haplotypes of *P*_*n*_. Since, the difference between *P*_*p*_ and *P*_*n*_ is only one transfer, we can introduce differential calculation of haplotypes *H*_*i*_^*n*^, *i* ∈ {1,2} of next partition from the haplotypes of *H*_*i*_^*p*^, *i* ∈ {1,2} of present partition. For this purpose, the algorithm stores *N*_*j*_^1^(*C*_*i*_^*p*^) and *N*_*j*_^0^(*C*_*i*_^*p*^) values of the present partition. After a transfer these values will either remain same or be incremented or decremented by 1. That is why it is now possible to construct *H*_*i*_^*n*^, *i* ∈ {1,2} in *O*(*k*) time. To compute *E*(*P*_*n*_) from the haplotypes requires *O*(*mk*) time. Thus running time to compute the *E*(*P*_*n*_) as well as to compute Gain(*f*) is *O*(*mk* + *k*).

For each intermediate partition *P*_*i*_, *i* = 1,…, *n*, we need to compute Gain measures for *m* − *i* unlocked fragments to find the maximum one. The transfer of this fragments requires updating of *N*_*j*_^1^(*C*_*i*_) and *N*_*j*_^0^(*C*_*i*_), *i* ∈ {1,2} and *j* = 1,2,…, *k*. Hence, it also needs *O*(*k*) time to run. Finally, there will be *m* such transfer in each iteration and maximum *m* rollbacks. Thus each iteration will require *O*(*m*(*m*(*mk* + *k*) + *k*) + *mk*) ~ *O*(*m* ^3^ *k*) running time.

**Figure 3:**
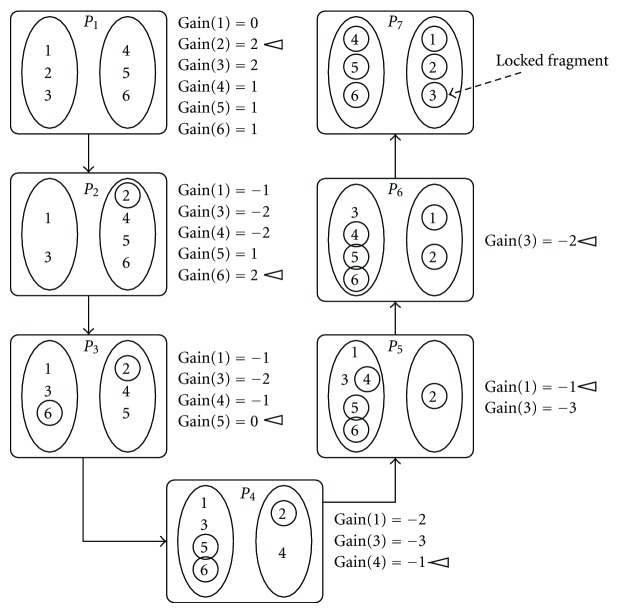
An example iteration of HMEC. The current partition *P*_*c*_ = *P*_1_ is the partition given in Figure 1(b) for the SNP matrix *M* of Figure 1(a). Locked fragments are indicated by circles.

**Figure 4:**
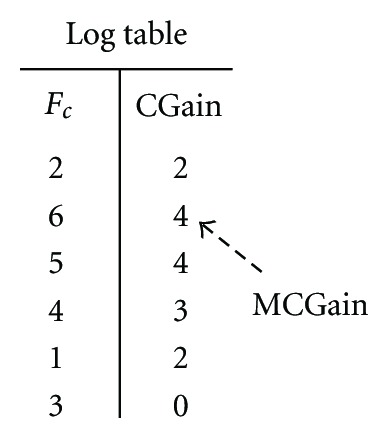
The log table corresponding to the iteration illustrated in Figure 3.

We now give an example illustrating a single iteration of our algorithm.  Figure 3 demonstrates an example iteration of HMEC. We consider that the current partition *P*_*c*_ = *P*_1_ is the partition given in Figure 1(b) for the SNP matrix *M* of Figure 1(a). All the intermediate partitions *P*_*i*_, *i* ∈ {1,…, 7} are shown sequentially and the gains of each fragment over the intermediate partitions are shown on the right of each partition. The free fragment with maximum gain is marked in each intermediate partition. For example, the fragment with the maximum gain in *P*_2_ is fragment 6 which has gain two. After each transfer, the transferred fragment is shown locked by a circle. Here, the ordering *F*_*c*_ of the fragments is 〈2,6, 5,1, 4,3〉 which is also the order of locking of the fragments. This order will be stored along with the *C*Gains in the log table.  Figure 4 demonstrates the resulting log table of the illustrated iteration. All the tentative transfers after *f*_max_ have to be rolled back so that the *P*_3_ becomes the next *P*_*c*_.

We now give an approximate gain measure to make our algorithm faster. For large SNP matrix, *O*(*m* ^3^ *k*) running time is critical to the performance of the algorithm. We can use an approximation in the calculation of the Gain(*f*) by using only the fragment *f* and not using the *m* − 1 other fragments. The approximate gain should be

(8) $$\begin{array}{c} \text{AppxGain}\left(f\right)=D\left(H_{i}^{p},f\right)-D\left(H_{j}^{n},f\right). \end{array}$$

Here *H*_*i*_^*p*^ is the haplotype of *f*'s present collection *C*_*i*_^*p*^ of partition *P*_*p*_, and *H*_*j*_^*n*^ is the haplotype of *f*'s next collection *C*_*j*_^*n*^ of partition *P*_*n*_. This function ignores the effect of fragments other than *f* on Gain(*f*), but reduces the run time of gain calculation to *O*(*k*). Therefore, the total run time of each iteration will be *O*(*m* ^2^ *k*).

## 5. Performance Comparison

In this section, we demonstrate the performance of our algorithm using both real biological and simulated datasets. We compared our algorithm with GMEC [5] and HapCUT [6]. We performed the simulation using the data from angiotensin-converting enzyme (ACE) [17] and public Daly set [18] to compare with GMEC [5], and used the HuRef data [19] to compare with HapCUT. We also compared HMEC with some other algorithms (SpeedHap [9], FastHare [10], MLF [11], 2 distance MEC [12], SHR-3 [13]) using ReHap website interface [15], which is an HapMap-based instance generator and comparison framework [14].

### 5.1. Comparison with GMEC

 In this section, we compare the performance of our algorithm with the genetic algorithm, which we call GMEC, described in [5].

We first sample the original haplotype pair into many fragments with different coverage and error rates. Each fragment works as a distinct sample of the same specimen. Here coverage rate indicates the percentage of the total columns of the SNP matrix that have been sampled out. The remaining slots are gaps. We then introduce some specific amount of error into these samples. The simulation was controlled in several ways. We varied the error rate while number of fragments and coverage rate were kept constant. Also, coverage was varied while number of fragments and error rate were kept constant.

Notice that the way we introduced error and controlled the coverage rate is not necessarily same as that of [5]. Therefore, the reconstruction rates of the branch and bound algorithm described in [5], which is an exact algorithm, should not be compared with those of HMEC.

#### 5.1.1. Experiment on Angiotensin-Converting Enzyme (ACE)

 Angiotensin-converting enzyme catalyses the conversion of angiotensin I to the physiologically active peptide angiotensin II, which controls fluid-electrolyte balance and systematic blood pressure. Because it has a key function in the renin-angiotensin system, many association studies have been performed with DCP1 (encode angiotensin-converting anzyme) [20]. Rieder et al. completed the genomic sequencing of the DCP1 gene from 11 individuals and reported 78 SNP sites in 22 chromosomes [17].

**Figure 5:**
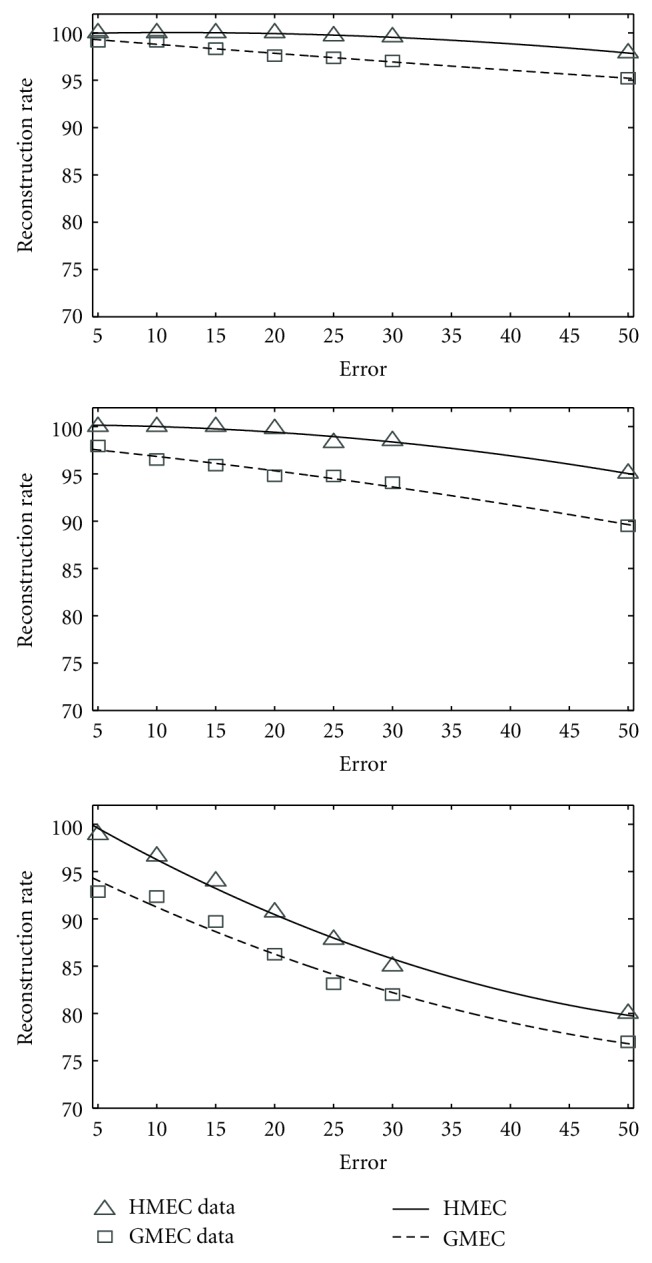
The comparison of HMEC and GMEC on ACE. From top to bottom, coverage = 75%, coverage = 50%, coverage = 25%.

**Figure 6:**
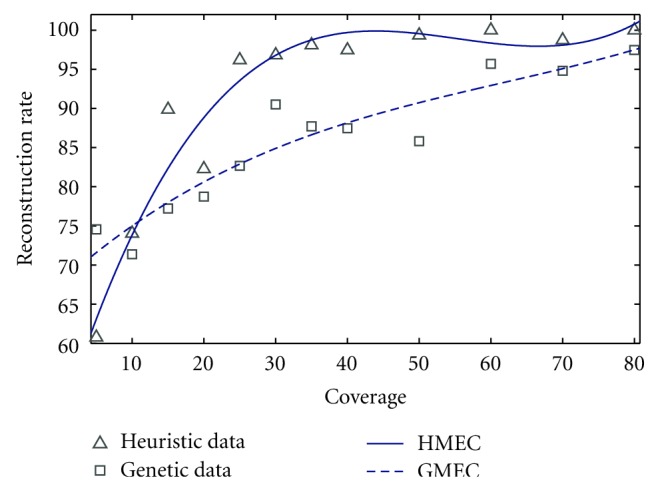
Reconstruction rate versus coverage value.

We take six pairs of haplotypes to perform the simulation. We generate 50 fragments from each of these haplotype pairs with varying coverage and error rate. We perform the simulation for three different coverage rates (25%, 50%, and 75%). For each of these coverage rates, we perform our simulation for different error rates such as 5%, 10%, 15%, 20%, 25%, 30%, and 50%. In every case, we compare our algorithm with GMEC.  Figure 5 illustrates the comparison that bears the clear testimony to the superiority of our algorithm. For most instances, the reconstruction rate achieved by our algorithm is 100% or greater than 98%. Only for a few cases with very high error rate and low coverage value, the reconstruction rate falls below 95%. We also perform the experiment for different coverage rates while keeping the error rate constant.  Figure 6 illustrates the performance of these two algorithms for various coverage values. Here also, our algorithm clearly outperforms the genetic algorithm except for very low coverage value (which is unrealistic).

#### 5.1.2. Simulation on Data from Chromosome 5q31

 In this section, we discuss our simulation results on the data from public Daly set. Daly et al. reported a high-resolution analysis of a haplotype structure across 500 kb on chromosome 5q31 using 103 SNPs in a European derived population which consists of 129 trios [18, 20].

**Figure 7:**
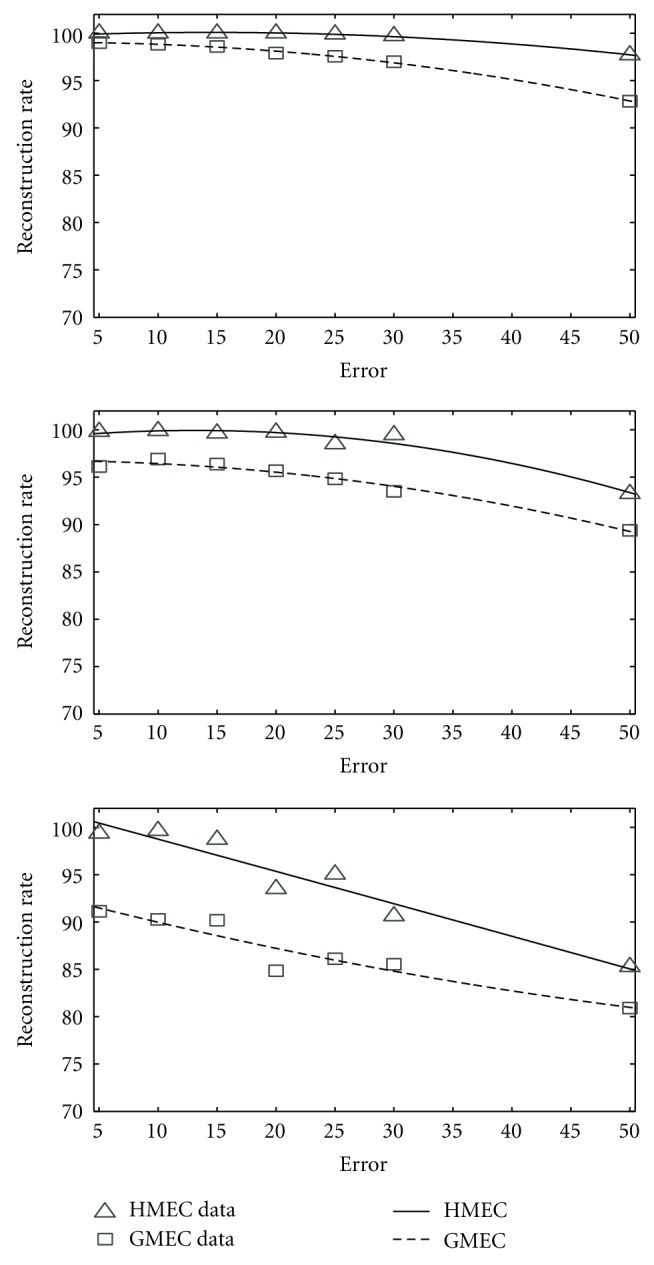
The comparison of HMEC and GMEC on Daly set. From top to bottom, coverage = 75%, coverage = 50%, coverage = 25%.

We performed the experiment exactly in the same way that we did for angiotensin-converting enzyme.  Figure 7 demonstrates the results. Experimental results suggest that HMEC is much better than the genetic algorithm. Again, for most cases, the reconstruction rate achieved by our algorithm is 100% or greater than 98%. For every instance, our algorithm exhibits better performance than that of GMEC.

#### 5.1.3. Experiment on Simulated Data

We used simulated data for further evaluation of HMEC. One of the very important advantages of our algorithm is that it takes very short time to reconstruct the haplotypes. Our algorithm is much faster than the GMEC.  Table 1 shows the running time of HMEC and GMEC. Here, we perform the simulation by varying the length (length denotes the number of SNP sites in the haplotype pair) of the haplotypes while fixed the value of the coverage rate and the error rate at 50%. Since haplotypes with such varying lengths are not available, we rely on the simulated data. Clearly, HMEC is much faster than GMEC. For example, while HMEC can reconstruct a haplotype with 936 sites in a fraction of a second, GMEC takes 72 seconds.

### 5.2. Comparison with HapCUT

HapCUT [6] is one of the most accurate heuristic algorithms for individual haplotyping. HapCUT uses a random initial haplotype configuration and builds a graph. It computes max-cut on the graph to find the position to flip and iterates until no improvement in MEC score is achieved. We have performed extensive experiments to compare the performance of HMEC and HapCUT. Experimental results suggest that although HapCUT is reliable, its running time is too large to be a realistic choice for whole genome haplotyping. Our algorithm computes the haplotypes significantly faster than HapCUT without losing accuracy.

We used the filtered HuRef data from Levy et al. [19] to evaluate the performance. We generated several test data sets varying the coverage and error rate, and tested the performance of HMEC and HapCUT on these data sets. The results are shown in Tables 2, 3, 4, and 5.

Experimental results indicate that the reconstruction rates of both HapCUT and HMEC are reasonably good. For very low coverage, reconstruction rate of HapCUT is slightly better than HMEC. However, as the coverage rate increases, HMEC begins to outperform HapCUT. Notice that HapCUT is only better than HMEC for very low (and thus unrealistic) coverage values (5% and 10% coverage). However, for higher coverage, HMEC consistently performs better than HapCUT. Furthermore, HapCUT is significantly slower than HMEC. For an instance, with 35% coverage and 40% error rate, HapCUT takes 215.5 seconds where HMEC takes only a fraction of a second (see Table 5). Therefore, although generally HapCUT provides reliable reconstruction rate, on large dataset, it is an unrealistic choice due to its time consuming operations. On the other hand, HMEC provides the high accuracy with much less amount of time.

We also used some sample data from ReHap project [14, 15]. We created the allele matrix from the ReHap error matrix and fed the matrix to both HMEC and HapCUT algorithms. HMEC consistently produces higher reconstruction rate than HapCUT (see Table 6). Also, the running time of HMEC is clearly much better than HapCUT.

### 5.3. Comparison Using ReHap

 We also compared HMEC with some other well-known algorithms such as SpeedHap [9], FastHare [10], MLF [11], 2 distance MEC [12], and SHR-3 [13] using HapMap-based instance generator and comparison framework [14, 15]. The results are shown in Table 7 and Table 8 that suggest that no single method clearly outperforms the others in all cases. However, reconstruction rates achieved by HMEC are the highest or very close to the highest. This bears a clear testimony to its suitability as a practical tool for individual haplotyping.

## 6. Conclusion

In this paper, we present a heuristic algorithm (HMEC) based on minimum error correction that computes highly accurate haplotypes significantly faster than the known algorithms for haplotyping. The algorithm is inspired from the famous Fiduccia and Mattheyses (FM) algorithm for bipartitioning a hyper graph minimizing the cut size [8]. We report on an extensive performance study evaluating our approach with other available techniques using both real and simulated datasets. Comprehensive performance study shows that our algorithm outperforms (in most cases) or matches the accuracy of other well-known methods, but runs in a fraction of the time needed for other techniques. High accuracy and very fast running time make our technique suitable for genome-wide scale data.

The accuracy of the algorithm can be improved by incorporating some prior knowledge. For example, small groups of fragments that are declared to be in the same haplotype can be identified. Probabilistic methods like expectation maximization (EM) also deserve some consideration over such optimization problems. In the near future, we intend to consider these issues to make further improvements.

## Figures and Tables

**Algorithm 1:**
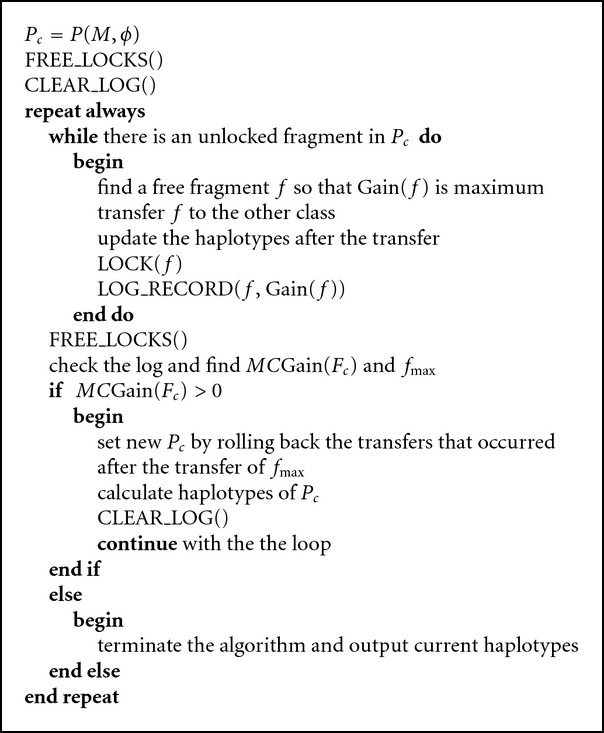
The HMEC Algorithm.

**Table 1:** Running time of HMEC and GMEC on simulated datasets.

Length of haplotype	HMEC	GMEC
	(sec)	(sec)
78	0.002	6.172
156	0.016	11.437
312	0.015	22.500
624	0.031	45.328
780	0.031	60.766
936	0.047	72.343

**Table 2:** Reconstruction rate and running time of HapCUT and HMEC with 5% coverage on HuRef data.

Error rate	HapCUT		HMEC	
	Reconstruction rate	Time (sec)	Reconstruction rate	Time (sec)
10	0.986	40.582	0.859	0.119
15	0.913	38.282	0.889	0.125
20	0.875	46.324	0.811	0.129
25	0.902	47.101	0.827	0.130
30	0.840	39.196	0.802	0.125
40	0.694	44.792	0.665	0.129

**Table 3:** Reconstruction rate and running time of HapCUT and HMEC with 10% coverage on HuRef data.

Error rate	HapCUT		HMEC	
	Reconstruction rate	Time (sec)	Reconstruction rate	Time (sec)
10	0.988	76.081	0.966	0.141
15	0.954	90.206	0.948	0.154
20	0.953	81.169	0.957	0.144
25	0.952	74.924	0.923	0.142
30	0.951	81.828	0.893	0.177
40	0.934	69.748	0.814	0.169

**Table 4:** Reconstruction rate and running time of HapCUT and HMEC with 25% coverage on HuRef data.

Error rate	HapCUT		HMEC	
	Reconstruction rate	Time (sec)	Reconstruction rate	Time (sec)
10	0.988	184.642	1.000	0.156
15	0.988	171.698	1.000	0.167
20	0.988	134.406	0.998	0.163
25	0.988	133.966	0.998	0.155
30	0.987	136.572	0.996	0.172
40	0.985	171.334	0.960	0.161

**Table 5:** Reconstruction rate and running time of HapCUT and HMEC with 35% coverage on HuRef data.

Error rate	HapCUT		HMEC	
	Reconstruction rate	Time (sec)	Reconstruction rate	Time (sec)
10	0.988	181.025	1.000	0.158
15	0.988	132.434	1.000	0.127
20	0.988	231.989	0.999	0.105
25	0.987	210.040	0.998	0.101
30	0.988	217.292	0.996	0.102
40	0.987	215.533	0.995	0.100

**Table 6:** Reconstruction rate and running time of HapCUT and HMEC for ReHap samples.

Error rate	HapCUT		HMEC	
	Reconstruction rate	Time (sec)	Reconstruction rate	Time (sec)
0.2	0.870	20.343	0.985	0.035
0.3	0.865	21.109	0.970	0.030
0.4	0.840	20.106	0.945	0.035
0.5	0.790	19.549	0.865	0.050

**Table 7:** Comparison of different haplotyping techniques using ReHap. Min Len = 3, Max Len = 7, coverage = 8. Highest reconstruction rate for each error rate is shown in bold.

Error rate	SpeedHap	FastHare	MLF	2-Distance MEC	SHR	HMEC	HapCUT
0.2	**1 **	0.91	** 1 **	** 1 **	0.65	** 1 **	0.9
0.3	0.935	0.7	0.945	0.61	0.685	**0.965 **	0.82
0.4	0.605	0.78	0.78	0.68	0.55	0.535	**0.81**
0.5	0.535	0.48	0.485	0.505	0.526	0.525	**0.58**

**Table 8:** Comparison of different haplotyping techniques using ReHap. Min Len = 10, Max Len = 30, coverage = 8. Highest reconstruction rate for each error rate is shown in bold.

Error rate	SpeedHap	FastHare	MLF	2-Distance MEC	SHR	HMEC	HapCUT
0.2	0.98	0.975	**0.985 **	** 0.985 **	0.84	** 0.985 **	0.87
0.3	**0.985 **	** 0.985 **	0.97	0.97	0.74	0.97	0.865
0.4	**0.895 **	0.79	0.885	0.885	0.675	0.87	0.84
0.5	0.74	0.47	0.76	0.76	0.51	0.705	**0.79**
